# Natural *Agrobacterium* Transformants: Recent Results and Some Theoretical Considerations

**DOI:** 10.3389/fpls.2017.01600

**Published:** 2017-09-13

**Authors:** Ke Chen, Léon Otten

**Affiliations:** ^1^Key Laboratory of Systems Biomedicine (Ministry of Education), Shanghai Center for Systems Biomedicine, Shanghai Jiao Tong University Shanghai, China; ^2^Institut de Biologie Moléculaire des Plantes, Centre National de la Recherche Scientifique (CNRS) Strasbourg, France

**Keywords:** *Agrobacterium rhizogenes*, *Nicotiana tabacum*, hairy roots, natural transformation, T-DNA

## Abstract

*Agrobacterium rhizogenes* causes hairy root growth on a large number of plant species. It does so by transferring specific DNA fragments (T-DNA) from its root-inducing plasmid (pRi) into plant cells. Expression of T-DNA genes leads to abnormal root growth and production of specific metabolites (opines) which are taken up by the bacterium and used for its growth. Recent work has shown that several *Nicotiana, Linaria*, and *Ipomoea* species contain T-DNA genes from *A. rhizogenes* in their genomes. Plants carrying such T-DNAs (called cellular T-DNA or cT-DNA) can be considered as natural transformants. In the *Nicotiana* genus, seven different T-DNAs are found originating from different *Agrobacterium* strains, and in the *Tomentosae* section no <4 successive insertion events took place. In several cases cT-DNA genes were found to be expressed. In some *Nicotiana tabacum* cultivars the opine synthesis gene TB-*mas2*′ is expressed in the roots. These cultivars were found to produce opines. Here we review what is known about natural *Agrobacterium* transformants, develop a theoretical framework to analyze this unusual phenomenon, and provide some outlines for further research.

## Introduction

*Agrobacterium* is well-known for its capacity to transfer part of its DNA to plants during a natural infection process leading to tumors (Crown galls) or abnormal roots (Hairy roots, HR) (Gelvin, 2012; Christie and Gordon, 2014; Kado, 2014). The genus *Agrobacterium* contains different species such as *A. tumefaciens, A. rhizogenes* (Riker, 1930), *A. vitis* (Ophel and Kerr, 1990), and *A. rubi* (Hildebrand, 1940). Another classification uses biotypes (Kerr and Panagopoulos, 1977). The transferred DNA (T-DNA) is located on a large plasmid (tumor-inducing or Ti plasmid) or root-inducing plasmid (pRi plasmid). Strains can carry one, two, or three T-DNAs on their pTi/pRi plasmid (Canaday et al., 1992). T-DNAs are surrounded by direct repeats of about 25 nucleotides (called borders). The transfer starts from the socalled right border and proceeds to the left border. Often, the integrated T-DNAs are incomplete and truncated at the left part. They can occur as single copies or as tandem or inverted repeats.

Genes located on the T-DNA are expressed in the plant cells and lead to growth changes (Binns and Costantino, 1998) and opine synthesis. Sterile Crown gall and HR tissues contain opines (Bielmann et al., 1960; Ménagé and Morel, 1964). They constitute different families of conjugated structures, the nature of which depends on the inciting bacterium. Opines often accumulate to very large quantities as they cannot be metabolized by the plant (Scott, 1979). Uptake and degradation of opines by *Agrobacterium* are encoded by specific genes located on the pTi or pRi plasmid, outside the T-DNA region(s), and agrobacteria can be attracted to opine sources by chemotaxis (Kim and Farrand, 1998). pTi/pRi plasmids can be transferred to other *Agrobacterium* strains by a conjugation process which can be induced by opines. Much has already been learnt about the way *Agrobacterium* transfers its T-DNA to plants (Gelvin, 2012; Christie and Gordon, 2014; Kado, 2014).

In 1983 it was discovered by Southern blot analysis (White et al., 1983) that *N. glauca* (Solanaceae family, *Noctiflorae* section of the *Nicotiana* genus) carries *A. rhizogenes*-like sequences in its nuclear genome. These sequences were called cellular T-DNAs (cT-DNAs). A more extensive study (Furner et al., 1986) involving other members of the *Nicotiana* genus revealed cT-DNA sequences in *N. tabacum, N. tomentosiformis, N. tomentosa*, and *N. otophora* (all belong to the *Tomentosae* section). Although *N. benavidesii* (section *Paniculatae*) was also mentioned as carrying a cT-DNA, there is no strong evidence for this.

A partial map of the *N. glauca* cT-DNA was obtained showing two dissimilar T-DNA copies linked together as an inverted repeat (called left and right arm). This map was later completed (Suzuki et al., 2002). In the case of *N. tabacum*, a few cT-DNA fragments were sequenced (Meyer et al., 1995; Fründt et al., 1998a,b; Intrieri and Buiatti, 2001; Suzuki et al., 2002; Mohajjel-Shoja et al., 2011). It has been reported that *C. arvensis* and carrot contain T-DNA sequences (D. Tepfer, cited in Matveeva and Lutova, 2014 and elsewhere), but this could not be confirmed by others (Matveeva and Lutova, 2014).

In 2012, a large-scale survey led to the discovery of cT-DNA sequences in *Linaria vulgaris*, a member of the Plantaginaceae family (Matveeva et al., 2012). In 2014, deep sequencing revealed four cT-DNAs (TA, TB, TC, and TD) in *N. tomentosiformis* and their distribution was studied in related species of the section *Tomentosae*. An additional type of cT-DNA sequence (TE) was found in *N. otophora* (Chen et al., 2014). In 2015, cT-DNA sequences were reported for *Ipomoea batatas* (Convolvulaceae family), a common crop. This species contains two cT-DNAs, *Ib*T-DNA1 and *Ib*T-DNA2. *Ib*T-DNA1 was found in cultivated sweet potatoes but not in wild relatives, whereas *Ib*T-DNA2 was found in both (Kyndt et al., 2015). Thus, gene transfer from agrobacteria to various plant species (natural genetic transformation) had occurred under natural circumstances. This led to genetically stable transformants, which we will call ≪ natural transformants ≫.

Although the study of natural transformants is still in its infancy, we would like to summarize recent observations and develop several theoretical considerations that may be useful for further investigations. We will start by having a close look at the agent that introduced the cT-DNAs: *A*. *rhizogenes*.

## *Agrobacterium rhizogenes* strains and their variability

Fründt et al. (1998a) speculated that cT-DNAs were initially normal plant sequences that were later captured by agrobacteria and employed for tumor and HR induction. We believe this is very unlikely because of the following reasons: cT-DNAs are absent from most plant species, their phylogenies do not match plant phylogenies, and the cT-DNAs end at the classical pRi T-DNA right borders as expected for transfer by *Agrobacterium*. Thus, there is little doubt that plants with cT-DNAs were indeed transformed by *Agrobacterium*.

The published cT-DNA structures all seem to be derived from *A. rhizogenes*-like T-DNAs. We know relatively little about *A. rhizogenes* strains, their Ri plasmids, and their T-DNA structures. Only a few strains have been studied and classified into mikimopine, cucumopine, agropine, and mannopine strains (represented by strains MAFF03-01724, NCPPB2659, ATCC15834, and NCIB8196 respectively) according to the opines they induce in the transformed roots. Their host ranges are very broad (De Cleene and De Ley, 1981).

The opine-based *A. rhizogenes* classification has no phylogenetic value because opine genes can be exchanged between different agrobacteria by horizontal gene transfer. Frequent horizontal gene transfer makes the construction of phylogenetic trees for T-DNA structures, pTi/pRi plasmids, and whole genomes practically impossible. Even if thousands of *Agrobacterium* genomes were available, it might still be impossible to establish phylogenetic trees (Van Nuenen et al., 1993). This was illustrated by a detailed analysis of *A. vitis*, the only *Agrobacterium* species for which a large number of isolates were compared. Three very different pTi types were found, but no intermediate structures, making it impossible to construct a tree. These studies suggested the selection of particular T-DNA gene combinations, loss of intermediates, and expansion of efficient strains into a few dominant groups (Burr and Otten, 1999).

Horizontal gene transfer also leads to chimeric T-DNAs. Examples are the pRi1724, pRiA4, and pRi2659 T-DNAs: their central parts are very similar, but close to the right border pRi1724 carries a mikimopine synthase (*mis*) gene, pRiA4 has an ornithine cyclodeaminase gene (*rolD*, Trovato et al., 2001), and pRi2659 a cucumopine synthase (*cus*) gene. These differences are most likely due to recombinations between different Ti plasmids (Otten and De Ruffray, 1994).

## Which types of *Agrobacterium* strains introduced the cT-DNAs?

Because pRi plasmids can be exchanged between *Agrobacterium* strains and are often chimeric, it is very difficult (if not impossible) to attribute a cT-DNA to a particular type of *Agrobacterium* strain. For example, the *N. glauca* cT-DNA strongly resembles part of the pRi1724 T-DNA, but the bacterium that introduced the cT-DNA is not necessarily derived from a 1724-like *A. rhizogenes* strain, since the remaining genome might be completely different. Unless natural transformation can be directly observed to occur in nature (see below), it will be impossible to identify the strain responsible for a natural transformation event on the sole basis of a cT-DNA sequence. In order to get a better idea of the pRi and T-DNA gene repertoire of *A. rhizogenes*, more isolates will have to be investigated. The variation in *A. rhizogenes* T-DNA structures is probably quite large, as shown by the new cT-DNA sequences. In *N. tomentosiformis*, six previously unknown T-DNA genes were found: two (in TA and TD) are distantly related to *orf14*, one codes for a protein with weak similarity to agrocinopine synthase (Acs, TB), another for a protein with weak similarity to octopine synthase (Ocs, TC), one for a C-like protein (*c*-like gene, TC), and one for a large, completely unknown protein (Orf511, TD). It is noteworthy that octopine synthase-like genes are normally only found in *A. tumefaciens* or *A. vitis*. In *N. otophora*, vitopine synthase (*vis*)-like sequences (distantly related to *ocs*) and *6b* genes with low similarity to their counterparts in *A. tumefaciens* and *A. vitis* were found alongside typical *A. rhizogenes* T-DNA genes such as *rolC, orf13*, and *orf14* (Chen et al., 2014). *Ib*T-DNA2 of *I. batatas* carries typical *A. rhizogenes* genes (*orf13, orf14, rolB, orf17n, orf18*) but with an unusual organization and an unusual *rolB*-like gene. *Ib*T-DNA1 carries *iaaM, iaaH*, C-protein, and *acs* genes (Kyndt et al., 2015). The latter gene combination has been found in *A. tumefaciens* strain C58 and in the *A. vitis* strain Tm4 TB region (Otten et al., 1999), but not in *A. rhizogenes*. These unusual T-DNA structures and genes were introduced by unknown *Agrobacterium* strains which might possess unusual root-inducing properties. However, if transformation happened long ago, strains might have evolved toward other forms or disappeared altogether.

In the next three sections we will discuss when the different transformation events could have taken place and how they relate to the evolutionary history of the recipient plants.

## Accumulation of cT-DNAs by successive transformations

When it was discovered that different *Nicotiana* species carry cT-DNAs in their genomes (Furner et al., 1986), it was suggested that this could result from the transformation of a common ancestor species. In a later report, two possibilities were proposed to explain the presence of T-DNA genes in *N. glauca* (*Noctiflorae* section, but at that time considered part of the *Paniculatae* section) and *N. tomentosiformis* (*Tomentosae* section). First, a T-DNA was inserted in an ancestor of these sections (part of the *Nicotiana* Cestroid ancestral complex) and inherited by the descendants. Second, the two cT-DNAs were inserted separately and independently, after the split between the two sections (Meyer et al., 1995). When the genome sequences of *N. tomentosiformis* (Chen et al., 2014), *N. otophora*, and three cultivars of *N. tabacum* (Sierro et al., 2014) became available, the situation turned out to be considerably more complex. The *N. tomentosiformis* genome was found to contain four cT-DNAs, each from a different *Agrobacterium* strain and different from the *N. glauca* cT-DNA. A fifth cT-DNA (TE) was discovered in *N. otophora* (*Tomentosae* section); its structure has not yet been assembled. The unexpected presence of related genes located on different cT-DNAs (such as the three *orf14* genes of TA, TB, and TD in *N. tabacum*) implied that phylogenetic analysis of partial cT-DNA sequences from different species (Intrieri and Buiatti, 2001) can only be carried out after it has been established whether they belong to the same cT-DNA or not.

**Figure 1:**
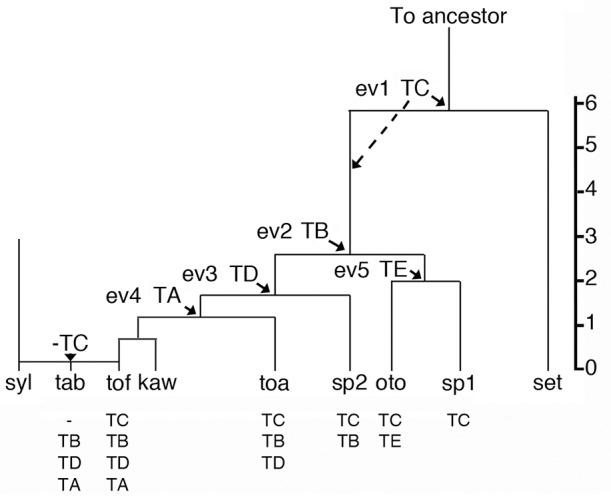
Phylogenetic tree of the *Nicotiana Tomentosae* section. The *Tomentosae* ancestor (To ancestor) splits into different groups. Arrows: arrivals of cT-DNA sequences (in the order TC > TB > TD > TA and TC > TE), here shown at at the separation of the branches. Alternatively, cT-DNAs could have arrived after the speciation events (indicated as an example for TC by dotted line). sp1 and sp2 represent hypothetical species. Vertical scale: % divergence between cT-DNA repeats. The *Tomentosae* tree based on cT-DNA insertions corresponds to the tree proposed by Knapp et al. (2004). syl, *sylvestris*; tab, *tabacum*; tof, *tomentosiformis*; kaw, *kawakamii*; toa, *tomentosa*; oto, *otophora*; set, *setchellii*; ev, insertion event. Below each species: cT-DNA content.

If one assumes that the four *N. tomentosiformis* inserts were introduced by successive transformations (and did not accumulate through crosses between different transformants), five different types of plants can be expected (Figure 1). In the *Tomentosae* section, the relative order of the insertion events (ev1 to ev4) can be estimated from the divergence values of the cT-DNA repeats (Chen et al., 2014, Table 1). Events 1, 2+3 (probably in the order TB > TD because of the differences in the repeat divergence), and 4 correspond to the introduction of TC, TB+TD, and TA. *N. setchellii* probably lacks a cT-DNA, as shown by the fact that its transcriptome contains no cT-DNA sequences (Long et al., 2016). *N. otophora* has two cT-DNAs (TC and TE, the latter being specific for *N. otophora* and introduced at event 5), *N. tomentosa* three (TC, TB, and TD), *N. kawakamii* and *N. tomentosiformis* four (TC, TB, TD, and TA). *N. tabacum* has three cT-DNAs, but its TC region has been completely deleted (including 1 kb of flanking DNA on each side, Chen et al., 2014). The remarkable loss of TC in *N. tabacum* shows the importance of investigating cT-DNA insertion sites (Chen et al., 2014; Chen, 2016). According to Figure 1, two intermediate *Nicotiana* forms (sp1 and sp2) are lacking in the *Tomentosae* section: one with TC, but without TE, and one with TC and TB (Figure 1). Possibly, they do occur as variants of existing species, as yet undetected species, or became extinct.

**Table 1:** Sequence divergence between repeats within different cT-DNA structures.

	**cT-DNA**	**Accession**	**% Divergence**	**References**
*N. tomentosiformis*	TA	KJ599826	1.2	Chen et al., 2014
	TB	KJ599827	2.6	Chen et al., 2014
	TC	KJ599828	5.8	Chen et al., 2014
	TD	KJ599829	1.7	Chen et al., 2014
*N. glauca*	Ng	AB071334.1 AB071335.1	3	Suzuki et al., 2002
*Ipomoea batatas*	*Ib*T-DNA1	KM052616.1 KM113766.1	0.9	Kyndt et al., 2015
	*Ib*T-DNA2	KM052617.1	0.7	Kyndt et al., 2015
*Linaria vulgaris*	T-DNA	EU735069	8.5	Matveeva et al., 2012

## Use of cT-DNA inserts as markers to reconstruct *Nicotiana* evolution

Transferred DNA (T-DNA) insertion events provide interesting clues to reconstruct plant evolution. All species with a cT-DNA at the same insertion site derive from a common ancestor in which the original insertion took place. The divergence between the repeats of such shared cT-DNAs should be consistent with the overall genome divergence between the species, but this has still to be tested.

Gemini viruses such as Geminivirus-Related DNA sequence (GRD, Murad et al., 2004) or retrotransposons such as the TS retrotransposons in tobacco (Wenke et al., 2011) can also provide clues for plant evolution. In the case of the *Tomentosae* section, it may be possible to date the different insertion events, since *Nicotiana* evolutionary trees have been calibrated, with an estimated DNA divergence of about 28% per 5 Mio years (Clarkson et al., 2005). The most diverged *Nicotiana* cT-DNA (TC) shows 5.8% divergence between the repeats which leads to an estimated age of 1 Mio years.

## cT-DNAs and evolution of *Ipomoea* and *Linaria*

In the case of *Ipomoea, orf13* sequences (from *Ib*T-DNA2) were detected in *I. batatas* and in *I. trifida* (Kyndt et al., 2015). This suggests that as in *Nicotiana*, cT-DNAs were introduced in an ancestor species and transmitted across speciation events. However, *Ib*T-DNA2 could have been transferred by interspecific hybridization, known to occur between *I. batatas* and *I. trifida* (Rouillier et al., 2013). Whether *Ib*T-DNA1 and *Ib*T-DNA2 were introduced by one or two transformation events is not clear, because both could be derived from a single *Agrobacterium* strain. The origin of the cultivated hexaploid (6x) species *I. batatas* is much debated. Two independent origins have been proposed which led to the socalled Northern and Southern lineages. The 6x genome has probably arisen in two steps, from 2x to 3x or 4x, and then to 6x. Possibly, *I. trifida* contributed to *I. batatas*, but it has also been proposed that *I. batatas* is derived from wild polyploid *I. batatas* plants (Rouillier et al., 2013). The distribution of cT-DNAs within *I. batatas* (both cultivated and wild forms) and *I. trifida* could shed new light on these questions. For *Linaria*, a calculation has been made on the basis of sequence divergence between *orf14-mis* sequences of *L. vulgaris, L. dalmatica*, and *L. acutiloba*. Assuming that the *orf14-mis* sequences are located on the same cT-DNA insert, the insert was estimated to be 1 Mio years old (Kovacova et al., 2014).

In none of the known cases, cT-DNA repeat divergence is more than 10% (see Table 1). This may indicate that cT-DNA insertions did not occur earlier than 1.5 Mio years ago. Alternatively, it may be that within this time span, the statistical probability of a complete cT-DNA deletion became sufficiently high, so that more diverged structures had little chance to survive.

## Could cT-DNA insertions lead to plant speciation ?

It has been proposed that cT-DNA insertions may have led to new species (Martin-Tanguy et al., 1996; Fründt et al., 1998a; Chen et al., 2014). In the case of the *Nicotiana Tomentosae* section different cT-DNA combinations were found in different species, and the order of cT-DNA entry corresponds to the proposed branching order of the species (Knapp et al., 2004; Chen et al., 2014, Figure 1). This pattern is consistent with the idea of speciation by transformation. Speciation could be favored by the strong effects of *A. rhizogenes* T-DNA genes on development (for example by changing flower morphology or flowering time), but this has not been investigated for natural or artificial HR transformants. The speciation hypothesis can be tested by comparing normal plants with their HR transformants obtained from *A. rhizogenes* infection under laboratory conditions. If indeed HR plants no longer hybridize with the ancestor and therefore have become new species, further studies could be carried out to identify the T-DNA genes responsible for introducing the change that leads to the reproductive barrier. Alternatively, cT-DNA sequences of natural transformants may be removed by CRISPR and the resulting plants compared with the unmodified natural transformant. However, the function of those genes that led to a reproductive barrier at an early stage might have been lost in later steps.

In the next section we will investigate in more detail what is known about the structures of cT-DNAs and their evolution.

## Structural organization of cT-DNAs

**Figure 2:**
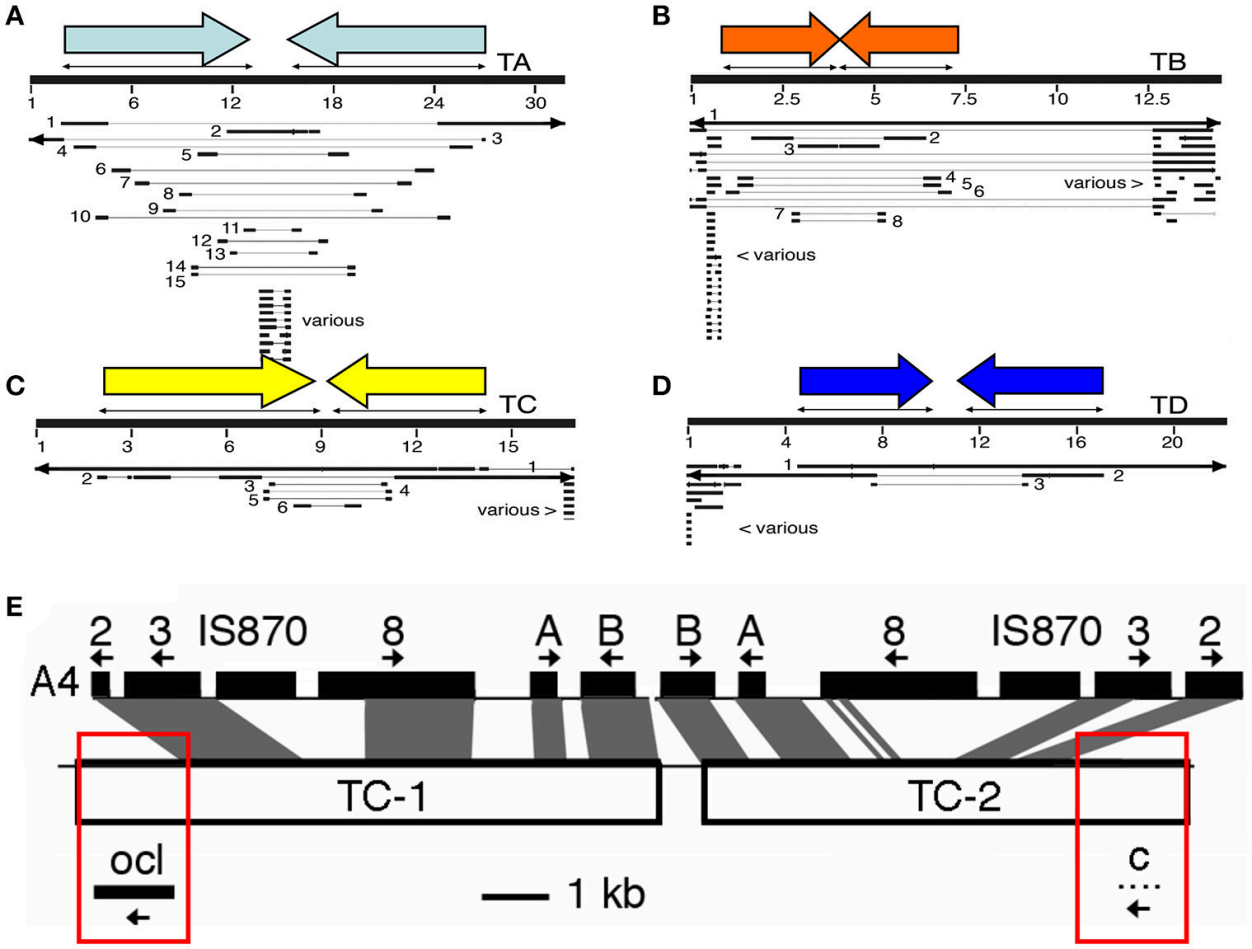
Maps of the four cT-DNAs from *N. tomentosiformis*. **(A–D)**: TA, TB, TC, and TD. Inverted repeats are indicated by colored arrows. Renumbered contigs from *N. tomentosiformis* (AWOL series, Sierro et al., 2014) are mapped on the final cT-DNA maps. The large numbers of contigs are due to difficulties in automatic assembly for these closely related inverted repeats. Various: repeated plant sequences, only part of these contigs are shown. **(E)** An example of a cT-DNA, the TC region from *N. tomentosiformis*. The figure shows the similarity between the rpeated part of TC and part of the *A. rhizogenes* A4 region with its T-DNA genes (A, B, C: *rolA, rolB, rolC*). Boxed in red are sequences (*ocl:* octopine synthase-like, and gene *c*) that have so far only been found in *A. tumefaciens* or *A. vitis*. Thus, the *A. rhizogenes* strain that inserted the TC-region belongs to a new type of strain that combines features of *A. rhizogenes* and *A. tumefaciens* or *A. vitis*.

In 8 out of 9 cases, cT-DNA structures are partial inverted repeats, inserted in a single site. The *Linaria* cT-DNA is an exception, being a partial direct repeat (Matveeva et al., 2012). In Figures 2A–D the four *N. tomentosiformis* cT-DNAs (TA, TB, TC, and TD) are shown with the original contigs constructed from small reads obtained by deep sequencing. Highly similar repeats can cause problems for the assembly of reads into contigs. This leads to many small contigs which must be linked by PCR amplification and sequencing. In Figures 2A–D the published *N. tomentosiformis* contigs (Sierro et al., 2014, AWOL series, renumbered) are shown aligned with the four assembled cT-DNA sequences. The TC region is shown in more detail (Figure 2E). The inverted repeat of TC partially aligns with TL from *A. rhizogenes* strain A4. At both ends of the repeat unique regions are found with an *ocl* gene on the left and a protein-C gene on the right. The T-DNA that gave rise to the TC region is unknown, and it is unclear how the inverted repeat and the single copy fragments were assembled. Further progress may require identification of *A. rhizogenes* strains with the relevant T-DNA genes.

All cT-DNAs seem to be truncated. In experimental infections with present-day *Agrobacterium* strains, T-DNA insertions can occur in different ways: in single sites (with a complete or truncated T-DNA, with direct or inverted repeats, with complete or incomplete repeats) or in multiple sites (with combinations of different structures). Some strains carry two different T-DNAs on their Ti/Ri plasmid, such as the TL and TR regions of *A. rhizogenes* strain A4 (Bouchez and Tourneur, 1991) and can introduce them separately or combined as a single insert. Potentially, this leads to a large variety of cT-DNA structures. The fact that most natural transgenic plants carry a single insert consisting of a partial inverted cT-DNA repeat is therefore probably not coincidental. No simple hypothesis can be proposed why this is so, but the following factors might be considered. cT-DNA inserts in multiple sites will segregate during sexual propagation, favoring single inserts. Repeat structures are more tolerant to mutations, thus facilitating preservation of important genes. Because T-DNA transfer starts at the right border and proceeds to the left, incomplete T-DNA structures will tend to have intact right borders and break off on the left. Studies on experimentally obtained regenerants or with additional natural transformants may show whether some structures are indeed preferred and what could be the underlying reasons.

In the next section we will discuss cT-DNA evolution and variability.

## Evolution of cT-DNAs

After stable integration, cT-DNAs will evolve through point mutations, insertions, and deletions, in the same way as normal plant DNA. Many cT-DNA genes in natural transgenic plants are interrupted by stop codons or are partially deleted (Table 2, see also below). Ng*rolB* of *N. glauca* is inactive but was converted to an active form by removal of two stop codons (Aoki, 2004). However, it is not clear whether the active form really corresponds to the original *rolB* gene. As expected, cT-DNA sequence variation can also occur within the same species. In early Southern blot experiments, cT-DNA variants were reported for *N. glauca* (Furner et al., 1986). Among *N. tabacum* cultivars, three TA variants occur (Chen et al., 2014).

**Table 2:** cT-DNA genes in different natural transformants.

**Species**	**cT-DNA**	**cT-DNA genes**	**Intact**	**Expr**.	**Biol. activity**	**References**
*Nicotiana glauca*	T-DNA	*rolB*	−	+	+ (after restoration)	Aoki and Syono, 1999c
		*rolC*	+	+	+	Aoki and Syono, 1999c
		*orf13*	+	+	+	Aoki and Syono, 1999b
		*orf14*	+	+	+	Aoki and Syono, 1999b
		*mis*	+	+	+ (in *E. coli*)	Suzuki et al., 2002
*N. tomentosiformis* and *N. tabacum* (tob)	TA	*orf8*	−	nt	nt	Chen et al., 2014
		*rolA*	−	nt	nt	Chen et al., 2014
		*rolB*	−	nt	nt	Chen et al., 2014
		*rolC*	+	+	+ (tob)	Mohajjel-Shoja et al., 2011
		*orf13*	+	+	+ (tob)	Fründt et al., 1998b
		*orf14*-like	−	+	nt	Chen et al., 2014
		*mis*	−	nt	nt	Chen et al., 2014
	TB	*orf14*	+	nt	nt	Chen et al., 2014
		*mis*	−	nt	nt	Chen et al., 2014
		*ags*	−	nt	nt	Chen et al., 2014
		*mas1'*	−	nt	nt	Chen et al., 2014
		*mas2′*	+	+	+ (tob)	Chen et al., 2016
	TC	*ocs*-like	+	nt	−	Chen et al., 2014
		*2*	−	nt	nt	Chen et al., 2014
		*3*	−	nt	nt	Chen et al., 2014
		*8*	−	nt	nt	Chen et al., 2014
		*rolA*	−	nt	nt	Chen et al., 2014
		*rolB*	−	nt	nt	Chen et al., 2014
		Gene *c*	−	nt	nt	Chen et al., 2014
	TD	*orf18*	−	nt	nt	Chen et al., 2014
		*orf14*-like	+	nt	nt	Chen et al., 2014
		*orf15*	+	nt	nt	Chen et al., 2014
		*orf511*	?	nt	nt	Chen et al., 2014
	TE	*vis*	?	nt	nt	Chen et al., 2014
		*6b*	?	nt	nt	Chen et al., 2014
		*mas1'*	?	nt	nt	Chen et al., 2014
		*mas2′*	?	nt	nt	Chen et al., 2014
		*rolB*	?	nt	nt	Chen et al., 2014
		*rolC*	?	nt	nt	Chen et al., 2014
		*orf13*	?	nt	nt	Chen et al., 2014
		*orf14*	?	nt	nt	Chen et al., 2014
		*iaaH*	?	nt	nt	Chen et al., 2014
		*iaaM*	?	nt	nt	Chen et al., 2014
		*acs*	?	nt	nt	Chen et al., 2014
*Linaria vulgaris*	T-DNA	*acs*	−	nt	nt	Matveeva et al., 2012
		*orf2*	−	nt	nt	Matveeva et al., 2012
		*orf3*	−	nt	nt	Matveeva et al., 2012
		*orf8*	−	nt	nt	Matveeva et al., 2012
		*rolA*	−	nt	nt	Matveeva et al., 2012
		*rolB*	−	−	nt	Matveeva et al., 2012
		*rolC*	+	−	nt	Matveeva et al., 2012
		*orf13*	−	−	nt	Matveeva et al., 2012
		*orf14*	−	−	nt	Matveeva et al., 2012
		*mis*	−	nt	nt	Matveeva et al., 2012
*L. dalmatica*	T-DNA	*rolC*	+	nt	nt	Matveeva and Lutova, 2014
*Ipomoea batatas*	*Ib*T-DNA1	*acs*	+	+	nt	Kyndt et al., 2015
		Gene *c*	+	+	nt	Kyndt et al., 2015
		*iaaH*	+	+	nt	Kyndt et al., 2015
		*iaaM*	+	+	nt	Kyndt et al., 2015
	*Ib*T-DNA2	*orf14*	−	nt	nt	Kyndt et al., 2015
		*orf17n*	−	nt	nt	Kyndt et al., 2015
		*rolB*-like	+	+	nt	Kyndt et al., 2015
		*orf13*	+	+	nt	Kyndt et al., 2015
		*orf18*/*orf17n*	+	nt	nt	Kyndt et al., 2015
*Ipomoea trifida*	*Ib*T-DNA2	*orf13*	+	nt	nt	Kyndt et al., 2015

*Adapted from Matveeva and Lutova (2014) No distinction is made between copies on repeats of the same cT-DNA. Since the orf511 gene from the TD region has no equivalent in the databases, it is unknown whether it is intact. As N. otophora contigs and reads have not yet been assembled, it is still unknown whether there are intact cT-DNA gene copies or not in this species. nt, not tested*.

cT-DNA evolution in *Ipomoea, Linaria*, and *Nicotiana* might be influenced by interspecific hybridization. *I. batatas* hybridizes with *I. trifida* (its closest natural relative) under natural conditions, although probably with low efficiency (Rouillier et al., 2013). The *Ib*T-DNA2 genes of *I. batatas* and *I. trifida* (Kyndt et al., 2015) could have been transferred by interspecific crosses. This could also apply to *L. vulgaris* and *L. dalmatica*, both of which contain cT-DNA sequences (Matveeva and Lutova, 2014) and are known to hybridize (Ward et al., 2009).

Interspecific crosses can have other consequences for cT-DNAs. *N. tabacum* results from an interspecific cross between *N. sylvestris* and *N. tomentosiformis* accompanied by massive genome reorganization (Lim et al., 2004). Whether this reorganization follows certain rules and reproducibly leads to the loss of the TC region, might be investigated with artificial hybrids.

When trying to understand cT-DNA evolution, one needs to reconstruct the original structures. This might be attempted by comparing the sequences of the cT-DNA repeats, both within the same species and between related species, favoring variants which correspond to intact open reading frames, are expressed and show biological activity.

In the next section we will investigate the important question of cT-DNA gene expression and regulation.

## cT-DNA expression and regulation

Although some studies have described cT-DNA gene expression and regulation, this field is still at its beginning and much remains to be done. Table 2 contains a list of expressed cT-DNA genes. Expression patterns depend on the insertion site and on the regulatory properties of the promoters. Promoter properties can be measured in different ways, either directly by mRNA analysis, or by using reporter genes. In reporter gene constructs promoters are linked to genes for visible markers, such as β-glucuronidase (GUS, Jefferson, 1987). Although much research has been carried out on T-DNA gene promoters (Maurel et al., 1990; Capone et al., 1991, 1994; Leung et al., 1991; Yokoyama et al., 1994; Di Cola et al., 1997; Hansen et al., 1997; Handayani et al., 2005), these studies should be extended in order to get a more detailed description of tissue-specificity, and to identify the corresponding plant transcription factors. Since T-DNA genes of Ri plasmids are expressed in hairy roots, it can be expected that cT-DNA genes are also expressed in roots. However, the properties of their promoters could have evolved, especially if expression in other plant parts would provide some selective advantage. Expression studies show that several cT-DNA genes have maintained their expression patterns in natural transgenic plants (Table 2). How and why T-DNA/cT-DNA genes are regulated the way they are, will need more research on T-DNA/cT-DNA function in hairy roots and natural transformants. It will be important to study those promoter properties in the right context. *A. rhizogenes* T-DNA reporter genes have rarely been studied in hairy roots. Likewise, cT-DNA promoters should be studied in the corresponding natural transformants. However, there is a danger that promoter constructs interfere with the expression of the genes from which they are derived, either by gene silencing or by competing for transcription factors.

The expression of *N. glauca* cT-DNA genes have received special attention because of their possible role in tumor formation. Interspecific hybridization between *N. glauca* and *N. langsdorffii* leads to socalled GGLL plants that spontaneously form tumors. It has been proposed that the *N. glauca* cT-DNA genes play a role in the abnormal growth of these tumors. Expression of Ng*orf13* and Ng*orf14* (Aoki et al., 1994; Udagawa et al., 2004), and Ng*rolB* and Ng*rolC* (Nagata et al., 1995, 1996) is enhanced in tumor tissues, possibly by a kind of inverted gene dosage effect (Martin-Tanguy et al., 1996). Up to now it has not been demonstrated that *N. glauca* T-DNA genes are indeed required for tumourous growth. For this they will need to be silenced or removed.

Another cT-DNA gene regulation study involved the TB-*mas2*′ gene of *N. tomentosiformis* and *N. tabacum*. Most tobacco cultivars and their paternal ancestor *N. tomentosiformis* have low TB-*mas2*′ expression levels (LE cultivars), but a few show high expression levels (HE cultivars). HE cultivars do indeed produce the expected *mas2*′ product desoxyfructosylglutamine (DFG) and are the only known cases so far of natural transformants which synthesize opines (Chen et al., 2016). The TB-*mas2*′ promoter sequences from HE and LE cultivars are identical, and P*mas2*′-GUS constructs are highly expressed in *N. benthamiana* roots, suggesting that TB-*mas2*′ can be silenced and re-activated. Silenced tobacco lines carrying artificially introduced *mas* genes could be re-activated by 5-azacytidine (Van Slogteren et al., 1984), but this was not the case for TB-*mas2*′ in LE cultivars (Chen et al., 2016). Mendelian inheritance of the LE/HE phenotype (Chen et al., 2016) suggested that activation and silencing of TB-*mas2*′ are due to a *cis* element linked to the TB insert.

Once it is established that cT-DNA genes are actively transcribed in natural transformants it will be necessary to investigate their influence on plant growth and metabolism.

## Role and activity of growth-modifying genes in natural transformants

The most interesting question concerning natural *Agrobacterium* transformants is undoubtedly whether they are mere accidents of evolution (by-products of hairy roots as it were, without any selective advantage), or whether cT-DNA integration led to new plant types with particular advantages compared to the non-transformed ancestors (Tepfer, 1983; Meyer et al., 1995). Since at least some natural transformants produce opines, they could also be of advantage to agrobacteria, without special advantages to the plants (sse below).

At the moment of writing, no direct evidence exists for a particular role for any of the cT-DNA genes within their normal context. However, some indirect arguments clearly indicate that they could influence the growth of natural transformants. The T-DNAs from *A. rhizogenes* carry genes known to induce hairy roots and these roots can be regenerated into plants with characteristic phenotypes, called the hairy root or HR phenotype. HR plants generally have a short stature with short internodes and wrinkled leaves (Tepfer, 1990; Christey, 2001; Lütken et al., 2012). Enhanced root growth could possibly improve survival under dry conditions. Among the *A. rhizogenes* T-DNA genes, the ≪ root locus ≫ (*rol*) genes *rolA, rolB, rolC*, and *rolD* influence hairy root induction on *Kalanchoe daigremontiana* leaves (White et al., 1985), and *rolA, rolB*, and *rolC* are sufficient to induce roots on several species. The *rolB* and *rolC* genes belong to the *plast* gene family, a large family of mostly T-DNA-located genes which includes *orf13, orf14, 6a*, and *6b* (Levesque et al., 1988; Studholme et al., 2005). *rolB* has a more general meristem-inducing activity (Altamura et al., 1994; Koltunow et al., 2001). In addition, *rolB* induces necrosis in tobacco leaves (Schmülling et al., 1988; Mohajjel-Shoja, 2010). *orf13* has been considered to be non-essential for root induction although capable of stimulating HR induction by *rolABC* genes (Cardarelli et al., 1987; Capone et al., 1989; Aoki and Syono, 1999a). However, *orf13* expression in tobacco (Hansen et al., 1993; Lemcke and Schmülling, 1998), tomato (Stieger et al., 2004), and *Arabidopsis* (Kodahl et al., 2016) led to various growth changes up to extreme dwarfism in *Arabidopsis* (Kodahl et al., 2016). The *rolA* gene has strong morphogenetic effects (Dehio and Schell, 1993; Guivarc'h et al., 1996). Thus, expression of *rol* genes and *orf13* in natural transformants can be expected to influence their growth.

*Linaria, Ipomoea*, and *N. otophora* contain *iaaH* and *iaaM* genes. Together these encode indole acetic acid synthesis and could have been active in early stages of transformation. It is noteworthy that the *iaaM* and *orf8* (Lemcke et al., 2000) T-DNA genes carry a *rolB*-like part at the 5′ end and a bacterial *iaaM* part at the 3′ end (Levesque et al., 1988). Both can be separated and retain their function (Otten and Helfer, 2001; Umber et al., 2002, 2005). Thus, an intact *rolB* part in an otherwise mutated *orf8* or *iaaM* gene might still influence the growth of natural transformants.

Ng*orf13*, Ng*rolC, trolC*, and *torf13* are expressed in the corresponding *Nicotiana* species. When overexpressed in tobacco, Ng*orf13* leads to dark-green rounded leaves (Aoki and Syono, 1999b), Ng*rolC* (Aoki and Syono, 1999c), and *trolC* (Mohajjel-Shoja et al., 2011) to a dwarf phenotype and lanceolate, pale leaves, whereas *torf13* induces green callus on carrot disks (Fründt et al., 1998b). In natural transformants, *rolC, orf13, orf14* are frequently intact (Table 2).

It is generally assumed that each type of T-DNA/cT-DNA gene has a specific effect, so that a cT-DNA-located *rolC* gene will have the same activity as a T-DNA-located *rolC* gene. However, variants of a given gene type can encode different biological activities. The *rolB* genes from 1,855 and 2,659 are less dependent on auxin for root induction on carrot disks as *rolB* from A4 (Schmülling et al., 1993; Serino et al., 1994). Six different *6b* genes from *A. tumefaciens* and *A. vitis* differ in their capacity to induce tumors (Helfer et al., 2002). Thus, functional differences between a cT-DNA gene and a related T-DNA gene (as noted by Aoki and Syono, 2000) might result from differences between the model strain and the strain that introduced the cT-DNA, rather than from divergent evolution after transfer to the plant.

The oldest cT-DNA (from *Linaria*) has lost all open reading frames except Lv*rolC*, suggesting positive selection of this gene. Inactivation of the *rolC, orf13*, and *orf14* genes in various natural transformants are obvious targets for the future.

It is possible that some (or even most) cT-DNA genes only played a role in the initial transformation/regeneration event, by allowing HR regeneration and the establishment of a new species (see above). After that, they could have lost their function either because of detrimental effects (like dwarfing by *rolA* or *orf13*, or necrosis by *rolB*) or because they were selectively neutral. In that case cT-DNA gene inactivation would show no effects and could lead to the wrong conclusion that these genes had no function in the evolution of the natural transformants. If cT-DNA genes induce significant morphological changes in other plants upon strong and constitutive expression, their expression in natural transgenics will probably also lead to changes, although these might be more restricted.

In the case of the widely cultivated tobacco and sweet potato, cT-DNA structures and expression patterns could have been subjected to selection during domestication. This hypothesis can be tested by careful comparison between certain cultivars and their isogenic cT-DNA mutants.

## Role of opine synthesis genes in natural transformants

T-DNA/cT-DNA regions generally contain opine genes. Opines are conjugation products of common metabolites such as amino acids, α-keto acids, and sugars, and cannot be metabolized by plants. Often, opine enzymes use multiple substrates (as in the case of lysopine dehydrogenase, Otten et al., 1977) thereby potentially sequestering a large amount of metabolites which might affect plant growth. Thus, it is important to know where T-DNA/cT-DNA opine genes are expressed, and to what extent they are regulated. The *rolD* gene strongly inhibits growth of transgenic carrot (Limami et al., 1998). In tomato, it does not affect morphology (the reason for the difference with carrot is unknown), but flowering occurs earlier with increased numbers of flowers and fruits (Bettini et al., 2003). Opines in crown galls and hairy roots are assumed to be secreted, in order to make them available to the agrobacteria, but this important process has not been studied in detail. It is unknown whether there are specific mechanisms for opine secretion, and whether T-DNA/cT-DNA genes play a role in this. It has been proposed that the *A. tumefaciens 6a* gene (a member of the plast gene family) stimulates secretion of octopine and nopaline (Messens et al., 1985), but unfortunately this interesting study has not been followed up.

Additional genes such as gene *c* and *orf511* (coding for a large, 511 amino acid protein) also remain to be studied. Gene *c* from *A. tumefaciens* strain C58 has shoot-inducing properties (Otten et al., 1999). Interestingly, it is also found in organisms other than plants (see below).

The morphological effects of various cT-DNA genes (expressed to different extents in different tissues) add up in complex ways. For example, *rolA* and *rolB* gene are antagonistic in tomato (Van Altvorst et al., 1992). *rolA, rolB*, and *rolC* (Spena et al., 1987), and *rolB, rolC, orf13*, and *orf14* act synergistically (Nilsson and Olsson, 1997; Aoki and Syono, 1999b). It will therefore be a particularly challenging task to establish the contribution of each gene in the context of their combined expression in natural transformants. In addition, two *Agrobacterium* T-DNA genes which are also found in natural transformants, can produce growth effects at a distance: *orf13* (Hansen et al., 1993) and *6b* (Helfer et al., 2003). This means that their effects might extend beyond their domains of expression.

Apart from changing plant growth, cT-DNA gene expression may confer immunity to *Agrobacterium* by silencing incoming T-DNA (for an experimental example of such T-DNA silencing, see Escobar et al., 2001). However, in the *Tomentosae* section agrobacteria were able to re-infect already transformed species, arguing against this possibility.

We will now investigate the question whether cT-DNA gene expression in natural transformants could influence the growth and evolution of *Agrobacterium*.

## Does *Agrobacterium* benefit from natural transgenic plants ?

Natural transformants which synthesize opines could influence the growth and evolution of *Agrobacterium* (Chen et al., 2016). In HE tobacco cultivars (see above) TB-*mas2*′ is expressed at high levels in root tips, and leads to production of significant amounts of DFG, a well-known opine (Chen et al., 2016). DFG can be used by agrobacteria and other microbes (Moore et al., 1997; Baek et al., 2005), but it has not yet been tested whether the DFG of HE cultivars is secreted and whether is might accumulate in the rhizosphere. Studies on artifical symbiosis based on opine utilization (Guyon et al., 1993; Dessaux et al., 1998; Savka et al., 2002; Mondy et al., 2014) provide experimental models to test this idea. Controlled inoculation of HE cultivars and isogenic CRISPR mutants with DFG-metabolizing and non-metabolizing *Agrobacterium* mutants could show whether DFG production by HE cultivars confers a selective advantage on DFG-using bacteria. If so, this could have some interesting implications. It has been postulated that the genetic modification of plant cells allows *Agrobacterium* to take control of its host, by re-directing its growth and metabolism to its own benefit. This process has been called ≪ genetic colonization ≫ (Schell et al., 1979). If it could be shown that opine production by HE plants favors *Agrobacterium* growth it would take the genetic colonization theory one step further. In that case the role of the pRi plasmid is not only (or even not at all) to induce hairy roots, but to create transgenic plants. Such plants could provide a genetically stable and much increased opine production, as compared to opine synthesis by relatively small numbers of non-permanent hairy roots growing from infected plants. If *Agrobacterium* benefits from opine production by natural transformants, hairy roots might be considered as mere intermediates on the way to transgenic plants. Opine production might be detrimental to plant growth, but reproductive isolation of the initial transformants could ensure their survival. Subsequently, cT-DNA functions might be selected against and growth might revert to normal. Thus, natural transformants could be transient plant species with various levels of genetic stability.

So far, it is not known how much *A. rhizogenes* benefits from opines produced in hairy roots growing in nature. Opine sources can attract Agrobacteria (Kim and Farrand, 1998) *in vitro*, but do agrobacteria also accumulate and multiply on hairy roots or on roots of natural transformants? What are the dynamics of these interactions? Do the bacteria concentrate around areas of highest production? Are opines stable in soil and do they accumulate over time? Do the modified growth properties of hairy roots increase opine production or secretion (for example by stimulating lateral root formation)? Experimental HR induction is generally done by infecting stems in the greenhouse or leaf disks *in vitro*, and the hairy roots develop in agar or in air. It would be interesting to know how hairy roots grow in soil and whether their growth is favored over that of normal roots. All these questions merit attention when one considers the effects of opine-producing plants on agrobacteria.

Apart from TB-*mas2*′, other opine synthesis enzymes (encoded by *acs, vis, ocl, mis, rolD*) should be investigated for their opine synthesis properties. Different forms with different substrate preferences may exist, as in the case of octopine dehydrogenase (Ocs, Otten and Szegedi, 1985).

Unusual growth characteristics of hairy roots and HR-derived plants could stimulate growth of agrobacteria independently from opines, for example if some T-DNA genes favor secretion of common root metabolites. When exploring the structure, expression and biological function of cT-DNA genes, it should be realized that some of these genes could have played a role in the first steps of the transformation/regeneration processes and that these events are still unknown. In the next section we will therefore look at a possible scenario for the evolutionary origin of natural transformants.

## A scenario for the origin of natural transformants

The details of the origin of natural transformants are still unclear. Different types of *Agrobacterium* strains with different T-DNAs were involved, as mentioned above. These could have induced different types of hairy roots, depending on their cT-DNA genes. In general, it is assumed that individual hairy roots represent clones growing from a single transformed cell (Tepfer, 1984; McKnight et al., 1987). A particular *A. rhizogenes* strain may induce hairy roots with different T-DNA structures (complete or incomplete) and different gene expression levels depending on the insertion sites, which probably leads to different types of roots. It is often assumed that hairy roots represent a single, well-defined type of roots, but this seems highly unlikely in view of the many combinations of T-DNA genes and expression levels expected to occur in individual hairy root clones. The occurrence of different agrobacteria strains, each with their own combination of T-DNA genes, increases the problem of HR variability. *A. rhizogenes*-induced roots have not yet been systematically investigated in terms of growth rate, cell division, elongation, differentiation, and root branching patterns. Plants regenerated from HR have not only modified roots, but also aberrant, wrinkled leaves and stunted growth. The conspicuously wrinkled leaves of HR plants have not yet been analyzed at the developmental level. Possibly they result from changes in vascular development. We suspect that a whole gradient of HR phenotypes may exist and that the expression ≪ hairy root phenotype ≫ is an oversimplification. Detailed cellular analysis of HR plants carrying T-DNA genes with inducible promoters will be of great use to understand how T-DNA genes affect growth (for an example using the *6b* gene, see Pasternak et al., 2017).

In the case of the natural transformants, there could have been a selection for HR types with T-DNA gene combinations that allowed plant regeneration. Some genes could be detrimental to regeneration (possibly *rolA*: inhibition of flowering, Martin-Tanguy et al., 1996; and *rolB:* necrosis, Schmülling et al., 1988), whereas others might favor this process.

In the case of the *Tomentosae* section, plants carrying the first cT-DNA (TC, carrying *rolA* and *rolB* genes) may have acquired a better regeneration capacity compared to the non-transformed ancestor. Thus, when TC-carrying plants were infected with another *A. rhizoge*nes strain, the resulting hairy roots (carrying TC and TB) could more easily regenerate into plants, and the process could repeat itself several times. Tobacco plants transformed by *A. rhizogenes* A4 spontaneously formed shoots from roots when grown in pots, contrary to normal tobacco (Tepfer, 1984). We need more research on the shoot regeneration properties of hairy roots in different species, the role of the different T-DNA genes in this process, and the underlying molecular mechanisms.

**Figure 3:**
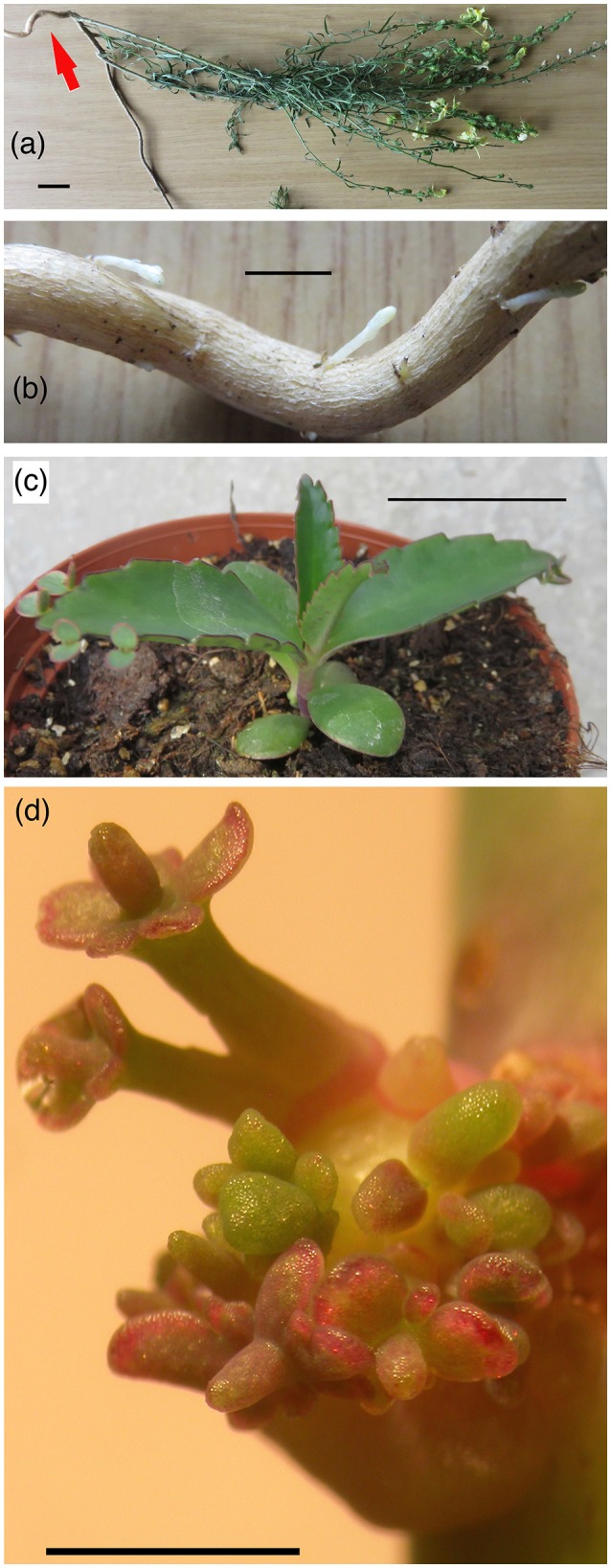
Regeneration of buds from *Linaria vulgaris* roots and of shoots from *Kalanchoe daigremontiana* tumors. **(a)** *L. vulgaris*, overview. Scale: 2 cm. **(b)** Detail buds. Scale 5 mm. **(c)** Normal *K. daigremontiana* plantlet. Scale: 3 cm. **(d)** Teratoma formation on *K. daigremontiana* stems infected with *A. tumefaciens* strain Tm4. The *Kalanchoe* teratoma structures are abnormal, but structured. Scale: 1 cm.

When considering the origin of natural transformants, it is worth noting that *A. tumefaciens* nopaline strains T37 and C58 (Yang and Simpson, 1981) or 82.139 (Drevet et al., 1994) can induce abnormal shoots (called shooty teratomas, Figures 3c,d). These are due to expression of the T-DNA-located isopentenyltransferase (*ipt*) gene, but shoot growth is probably also influenced by other T-DNA genes. It would be worth investigating whether teratomas could lead to rooting plants under natural conditions and eventually give rise to natural transformants.

Some plant species may have special regeneration abilities, so that hairy roots induced on such plants could easily produce fertile plants. *Linaria* carries buds on its roots, which may greatly facilitate plant regeneration from hairy roots (Figures 3a,b). *L. vulgaris* (but not *L. maroccana*) internode fragments easily form shoots and callus *in vitro*, even on hormone-free medium (Matveeva et al., 2012). It remains to be shown whether this is an intrinsic property of some *Linaria* species or due to cT-DNA genes. *I. batatas* shoot fragments (called slips) easily form roots, whereas root pieces carry dormant buds which easily produce plants (George et al., 2011). Re-transformation events may be favored if opine-producing plants attract agrobacteria. These could then introduce additional cT-DNAs (Chen et al., 2014).

In order to definitely establish themselves, the new transgenic plants had to transmit their cT-DNA to their progeny and reproduce successfully in the same environment as the ancestors. It is questionable whether a presumably very rare natural transgenic plant could have survived without reproductive isolation (sympatric speciation, see below). Later, the need for reproductive isolation might have disappeared, when sufficient differences had accumulated to prevent hybridization with the ancestral species. This could have led to the counterselection of cT-DNA genes that were important for speciation, especially if they reduced growth and reproduction. Selection to reduce negative cT-DNA effects could also have occurred elsewhere in the plant genome.

It is often assumed that natural transformants are homozygous for cT-DNA sequences, but it is possible that different cT-DNA gene alleles occur in natural populations (for intraspecific cT-DNA variants, see above). Selectively neutral genes would gradually be eroded and finally disappear. In extreme cases, the complete insert could be lost, as observed for the *N. tabacum* TC-DNA. TB-*mas2*′ seems to have been silenced in *N. tomentosiformis* and subsequently re-activated in some *N. tabacum* cultivars (Chen et al., 2016) which might constitute a case of evolutionary ≪ reversion ≫.

**Figure 4:**
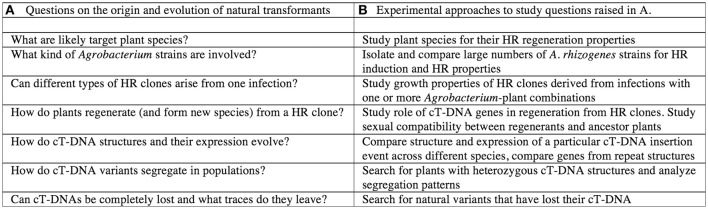
Theoretical steps in the origin of natural transformants. **(A)** Questions on the origin and evolution of natural transformants. **(B)** Experimental apporaches to study the questions raised in A.

Thus, to ensure the transition from a hairy root clone to the many successful populations of present-day natural transformants such as *Nicotiana glauca* or *Linaria vulgaris*, many steps might have been necessary. For a summary of these steps, see Figure 4A. The following section suggests some experiments to investigate this scheme (summarized in Figure 4B).

## Experimental evidence for evolutionary scenarios

What kind of experimental evidence could lend support to theoretical evolutionary scenarios as described above? It seems impossible to reconstruct the exact transformation events and the subsequent evolution leading to present-day natural transformants. However, if similar events still occur in nature, one might learn more about them. In the case of the natural *Nicotiana* transformants, it could be investigated whether *Nicotiana* species from the *Tomentosae* or *Noctiflorae* section are infected by *A. rhizogenes* in their natural South-American environment, and one could try to isolate and characterize *A. rhizogenes* strains from their rhizosphere.

The next question concerns the capacity of hairy roots to spontaneously produce transgenic plants under natural conditions. This may be studied by challenging different plant species with different *A. rhizogenes* strains under controlled conditions, preferably using plants growing in soil. Regeneration of plants from hairy roots under laboratory conditions has been reported for 53 plant species (Christey, 2001). However, nothing is known about conditions that favor regeneration in nature, such as climate, humidity, age of the plant, type of soil, type of wounding, or site of infection. Starting with a system of robust HR induction on plants growing in soil, it might be possible to study plant regeneration from such roots. Several ornamental plants have been transformed with natural *A. rhizogenes* strains in order to obtain dwarfed forms, a desirable trait in horticulture (Lütken et al., 2012). Such applied HR research could address several of the questions raised above (HR types, effects of cT-DNA genes, regeneration capacity, reproductive isolation). A significant potential exists for plant improvement using *A. rhizogenes* T-DNA genes (Christey, 2001; Casanova et al., 2005; Guillon et al., 2006) which probably also applies to cT-DNA genes.

In order to study possible ancestor phenotypes, cT-DNA genes might be silenced or removed by CRISPR. Compared to the CRISPR approach, silencing may have an interesting advantage: placed under control of an inducible promoter, a silencing construct could reduce expression of a target gene to different levels and in a spatially and temporally controlled way.

Naturally transformed plants have so far been found in the genus *Nicotiana, Linaria*, and *Ipomoea*. In the next part we will discuss how to search for additional transformants.

## Search for additional natural transformants

In order to search for natural transformants, three approaches can be used. First, deep sequencing of many plant species is yielding vast numbers of DNA sequences, both from genomic DNA and from transcriptomes. These sequences can be regularly analyzed for T-DNA-like sequences by automatic search robots. The cT-DNAs of the *Nicotiana* group have revealed the presence of genes that were thought to be specific for *A. tumefaciens* or *A. vitis* (*6b, ocl, vis*, Chen et al., 2014). Therefore, query sequences should not only include all known *A. rhizogenes* T-DNA sequences, but *A. tumefaciens* and *A. vitis* T-DNAs as well. In order to increase the chance of finding sequences with weak homology to model sequences, nucleotide data bases can be interrogated with protein query sequences (NCBI, tblastn search).

Second, plant species with close affinity to natural transformants or different accessions of the same species should be investigated, in order to define the distribution limits of the cT-DNA sequences within a group of species, and to explore their structural and functional variability.

Third, species that easily form plants from root fragments, have wrinkled leaves or other HR characteristics, might be candidates and could be tested by PCR experiments or deep sequencing.

We believe that the search for cT-DNA sequences should not be limited to plants. The capacity of *Agrobacterium* to introduce T-DNA genes into fungi under laboratory conditions has been well documented (de Groot et al., 1998; Michielse et al., 2008). It seems possible that this also occurs in nature, especially in the mycosphere (Zhang et al., 2014). Regeneration of transformed cells might be easy in such organisms, since single cells can be transformed. No *bona fide* cT-DNA sequences have yet been found outside the plant world. However, protein searches led to the discovery of several T-DNA-like protein sequences in fungi (Mohajjel-Shoja et al., 2011; Chen et al., 2014). Among these, opine enzyme-like sequences were found in *Nectria hematococca* (Acs), *Aspergillus nidulans* (Ocl), and Sus-like proteins are relatively widespread in various fungi. Plast proteins were detected in *Laccaria bicolor*. Protein C sequences were found in *Melampsora larici-populina* and *Pestalotiopsis fici* (Chen et al., 2014). These fungal T-DNA-like sequences are more divergent with respect to known T-DNA sequences than the plant cT-DNA *plast* sequences (Table 3) and could be derived from other types of *Agrobacterium* strains. Their patchy distribution among fungi argues in favor of horizontal gene transfer. Some fungi (such as *Pestatoliopsis* and *Melampsora*) contain several T-DNA-like genes. If such genes are grouped (as expected in the case of T-DNA transfer), this would provide a argument for ancient T-DNA transfer. Further investigations should concentrate on the chromosomal sequences around these genes and their comparison with relatives lacking such genes. Finally, it will be important to investigate their expression and function.

**Table 3:** T-DNA-like protein sequences in fungi.

**Protein category**	**Organism**	**Accession number**	**Closest T-DNA relative**	**Accession number of closest relative**	**% Identity**
Opine enzymes	*Nectria haematococca, Fusarium oxysporum*	XP_003047010.1 XP_018252422.1	ChsA (not on T-DNA), Ags	AAK08601.1 P27875.1	60 42
	*Aspergillus niger* and many others	EHA20957.1	TC-Ocl	XP_009611266	40
	*Melampsora larici-populina*	EGG11641.1	TC-Ocl	XP_009611266	34
Plast proteins	*Laccaria bicolor*	XP_001884962	Protein 5 (Tm4)	AAB41873	21
	*Laccaria bicolor*	XP_001884963	Protein 5 (Tm4)	AAB41873	20
	*Laccaria bicolor*	XP_001884964 (409aa, first part)	Protein 5 (Tm4)	AAB41873	19
	*Laccaria bicolor*	XP_001884861 (451aa, last part)	Protein 5 (Tm4)	AAB41873	22
	*Laccaria bicolor*	XP_001881215 (491 aa)	C' protein	NP_053417.1	19
C protein	*Melampsora larici-populina*	EGG11381.1	C (C58)	AAD30491.1	30
	*Pestalotiopsis fici*	XP_007840635.1 (540aa)	C (C58)	AAD30491.1	32

## Conclusions

Natural *Agrobacterium* transformants represent special cases of horizontal gene transfer, as they result from a highly adapted process aimed at the transfer and insertion of functional genes in plants. The bacteria responsible for the insertion of the cT-DNAs were probably related to *A. rhizogenes*. The natural variability of this bacterium and the capacity of various *A. rhizogenes* types to induce hairy roots in nature (and not only under laboratory conditions), both on aerial parts and in soil, is still largely unexplored. Spontaneous regeneration of natural hairy roots may depend on the properties of the non-transformed hosts, but probably also involves cT-DNA genes. More studies are required on the function and molecular mechanism of the T-DNA genes, in order to explain how and why natural transformants differ from their ancestors, and how they managed to establish themselves. An important direction for future research will be the removal or silencing of cT-DNA genes. The *plast* genes, opine genes, *rolA*, gene *c*, and *orf511* all require detailed analysis by themselves. Opine synthesis by natural transformants and its potential to favor *Agrobacterium* growth should be investigated under natural conditions, and should include studies on the influence of opine synthesis on plant metabolism, and on the mechanisms and specificities of opine secretion. The *plast* genes constitute an especially challenging subject as 30 years of research have not been able to convincingly reveal their basic function. They seem to be involved in the transport of plant metabolites and in the induction of abnormal growth. Studies on cell division and differentiation of various types of hairy roots and HR plants will be essential to understand how T-DNA/cT-DNA genes redirect the growth of roots and other plant organs. In view of their strong morphogenetic activities, both T-DNA and cT-DNA genes may be used for applications in horticulture and agriculture. Such research would undoubtedly benefit from a better understanding of their functions.

## Author contributions

LO wrote the basic structure of the paper. KC participated in writing and correcting the paper.

### Conflict of interest statement

The authors declare that the research was conducted in the absence of any commercial or financial relationships that could be construed as a potential conflict of interest.
